# Kinetics of Synaptic Protein Turnover Regulate Synaptic Size

**DOI:** 10.1371/journal.pbio.0040404

**Published:** 2006-11-07

**Authors:** Rachel Jones

When most of us picture a cell—or any subcellular component, like the nucleus—we often imagine a fairly static, solid entity. The molecules of the membrane and all the intracellular machinery fit together like pieces of a jigsaw puzzle. But in reality, the proteins, lipids, and other molecules that make up a cell and its parts are incredibly mobile and often short-lived.

In this unstable environment, how does the cell maintain and control its various functions? Karel Svoboda and colleagues have addressed this question by investigating how a protein called PSD-95 spreads within cells and how this transport and diffusion modulate the strength and size of neuronal connections. PSD-95 inhabits a compartment in neuronal synapses (the communication junction between neuron pairs) called the postsynaptic density, where the receptors that detect neurotransmitters released by a neighboring neuron are sited. PSD-95 helps to anchor these receptors in place. In certain types of synapses, the postsynaptic density caps the end of a specialized structure called a spine, which looks a little like a tiny mushroom sticking out from the cell membrane.

Synapses and spines can grow and shrink, and they appear and vanish throughout life, but others are stable and can last for months. However, the proteins that form essential structures in the postsynaptic density and spine, including PSD-95, last for only hours. Svoboda’s team set out to investigate the dynamics of clusters of PSD-95 and how they affect spine and synapse stability.

To be able to see spines in living brains, the authors introduced the genes for two proteins—a red fluorescent protein called mCherry, and PSD-95 tagged with a green fluorescent protein (GFP)—into neurons in embryonic mice. After the mice were born, Svoboda and colleagues removed a small piece of their skulls and replaced it with a tiny “window,” through which they could view the brain. Using a specialized technique called dual-laser two-photon laser scanning microscopy, they could see individual spines and the distribution of green fluorescent PSD-95. Within the spines, and particularly at their tips, green fluorescent buds (called puncta) represented clusters of PSD-95. These clusters did not seem to move, shrink, or grow over the course of a 90-minute imaging session. In some instances, these clusters were stable for days.

To investigate the behavior of individual molecules of PSD-95, the authors used a form of GFP that is normally not visible but can be “photoactivated” by a specific wavelength of light. After the photoactivation, bright fluorescence in the spines faded (over tens of minutes), showing that the photoactivated molecules of PSD-95 were leaving and, presumably, being replaced by nonphotoactivated molecules that entered the postsynaptic density from elsewhere. At the same time, fluorescence gradually appeared in neighboring spines, indicating that photoactivated PSD-95 was moving between spines. The time course of this turnover was much less than the lifetime of a spine or the half-life of PSD-95. While simple diffusion could predict how quickly PSD-95 exchanged between synapses, Svoboda and colleagues found that the rate of PSD-95 turnover within spines is mainly a function of its binding to other molecules in the postsynaptic density.

Large spines contain more PSD-95 than smaller ones and are also more stable. If the kinetics of PSD-95 at all synapses were identical, diffusion would eventually lead to all synapses containing the same amount of PSD-95. But this does not happen—larger postsynaptic densities capture more PSD-95 and retain it longer than smaller ones. Changes in size and changes in retention time are highly correlated. These synapse-specific kinetics could result from the effects of spine geometry or from biochemical mechanisms, such as differences in binding partners for PSD-95 between synapses of different sizes, or from synapse-specific regulation of post-translational modifications of PSD-95.

Synapses and spines become more stable with increasing age during development, and Svoboda’s team showed that the retention of PSD-95 in the postsynaptic density increases in parallel. But synapses are also highly plastic, responding rapidly to changes in synaptic activity. When the inputs to the area of cortex being studied were reduced—by trimming off the whiskers that provide sensory input to that part of the somatosensory cortex in mice—PSD-95 retention at the postsynaptic density dropped markedly, showing that the kinetics of PSD-95 turnover are experience-dependent.

These findings show that PSD-95 is shared between synapses and spines, and that the kinetics of PSD-95 turnover within spines are tuned to maintain the appropriate size of the postsynaptic density at each synapse. Attention will now turn to the mechanisms of this tuning and to the potential role of PSD-95 in contributing to synaptic plasticity.

